# Nail Gun Injury of the Trachea and Spinal Cord

**DOI:** 10.5811/cpcem.2022.3.56410

**Published:** 2022-07-27

**Authors:** Kohei Shibahashi, Kenji Numata

**Affiliations:** St. Marianna University School of Medicine, Department of Emergency and Critical Care Medicine, Kawasaki, Japan

**Keywords:** spinal cord injury, orthopedic, neurology, nail gun injury, emergency medicine

## Abstract

**Introduction:**

A 26-year-old man was impaled by a nail after a nail gun accident. He was fully conscious with weakness and loss of sensation in the extremities. Cervical computed tomography showed a 9-centimeter long nail penetrating the spinal cord. The nail was removed surgically six hours after the incident. Neurological deficits gradually improved, and at three-month follow-up the patient had completely recovered from muscle weakness and reported only mild sensory deficits in the bilateral sole of his foot.

**Discussion:**

This case showed a favorable neurological course, which may be attributed to the fact that the cervical spinal cord injury did not involve the corticospinal tracts and anterior horn.

## INTRODUCTION

**Image 1:**
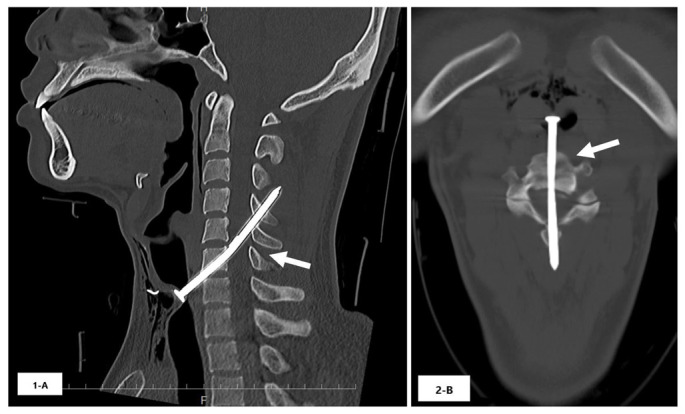
Cervical computed tomography showing a 9-centimeter nail penetrating the trachea, anterior vertebral body, and spinal cord (arrows).

The trachea and spinal cord of a 26-year-old man were penetrated by a nail after he slipped while using a nail gun and accidentally shot a nail into his neck. A 5-millimeter hole was found under the laryngeal ridge. His airway, breathing, and circulation were sustained. He was fully conscious with weakness in the extremities (manual muscle test scores of both upper and lower extremities were 3), and decreased sensation below the xiphoid level, as well as bladder and rectal disturbance. Cervical computed tomography showed a 9-centimeter nail penetrating the trachea, anterior vertebral body, and spinal cord (Image 1).

Because the nail had penetrated the trachea, a tracheotomy was performed by an otolaryngologist while the patient was conscious. The nail was removed under general anesthesia six hours after the incident by an orthopedic surgeon. His neurological deficits gradually improved, and at three-month follow-up he had completely recovered from muscle weakness and reported only mild sensory deficits of the bilateral sole of his foot.

## DISCUSSION

**Image 2:**
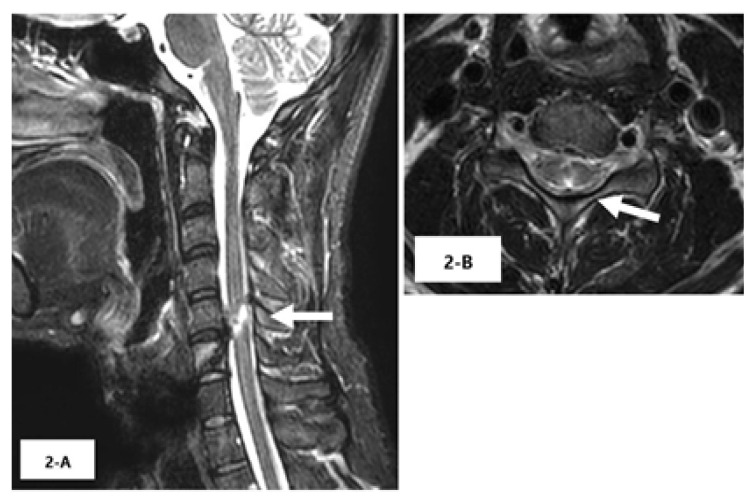
Postsurgical cervical magnetic resonance imaging showing high T2 signal intensity at the posteromedial region of the sixth cervical level, without any evidence of corticospinal tract damage (arrows).

The mechanism of injury in a patient with traumatic spinal cord injury can be divided into two major categories: blunt spinal cord injury (BSCI), and penetrating spinal cord injury (PSCI). Outcomes for patients with PSCI remain relatively understudied. However, Roach et al reported that PSCI results in poorer neurological and functional outcomes than BSCI, and only 19.6% patients with PSCI undergo spinal cord surgery (vs 80.6% for those with BSCI).^1^ They reported that this discrepancy may reflect the uncertainty of neurological benefits of surgery for patients with PSCI due to the lack of evidence for surgical treatment in this population. The present case showed a good neurological course. Postsurgical cervical magnetic resonance imaging revealed high T2 signal intensity at the posteromedial region of the sixth cervical level (Image 2).

Lateral corticospinal tracts and anterior horn are located on the sides of spinal cord. In our case, the good neurological course was believed to be attributed to the penetration of the nail to the center of the spinal cord; the cervical spinal cord injury did not involve the corticospinal tracts and anterior horn. 2 We considered that the nail penetrated the gracile fasciculus because this anatomic feature is at the center of the spinal cord. Therefore, the patient’s sensory disturbance was localized to his legs.

**CPC-EM Capsule**

What do we already know about this clinical entity?

*Traumatic spinal cord injury results in poor neurological and functional outcomes; only 19.6% of patients with penetrating spinal cord injury undergo spinal surgery*.

What is the major impact of the image(s)?

*These images of a 9-centimeter long nail that penetrated the patient’s spinal cord and resulted in muscle weakness suggest a poor neurological outcome*.

How might this improve emergency medicine practice?

*Non-involvement of the corticospinal tracts and anterior horn in penetrating spinal cord injuries may be a predictor of favorable neurological course following surgery*.
